# Proton minibeam radiation therapy widens the therapeutic index for high-grade gliomas

**DOI:** 10.1038/s41598-018-34796-8

**Published:** 2018-11-07

**Authors:** Yolanda Prezado, Gregory Jouvion, Annalisa Patriarca, Catherine Nauraye, Consuelo Guardiola, Marjorie Juchaux, Charlotte Lamirault, Dalila Labiod, Laurene Jourdain, Catherine Sebrie, Remi Dendale, Wilfredo Gonzalez, Frederic Pouzoulet

**Affiliations:** 1 Laboratoire d’Imagerie et Modélisation en Neurobiologie et Cancérologie (IMNC), Centre National de la Recherche Scientifique (CNRS), Universités Paris 11 and Paris 7, Campus d’Orsay, 91405 Orsay, France; 2 Institut Pasteur, Neuropathologie Expérimentale, Institut Pasteur, 28 Rue du Docteur Roux, 75015 Paris, France; 3 Institut Curie, PSL Research University, Radiation Oncology Department, Centre de Protonthérapie d’Orsay, 101, F-91898 Orsay, France; 4 Institut Curie, PSL Research University, Translational Research Department, Experimental Radiotherapy Platform, Orsay, France; 5 Paris Sud University, Paris -Saclay University, 91405 Orsay, France; 6 IR4M, UMR8081, Université Paris Sud, CNRS, Université Paris-Saclay, 91405 Orsay, France

## Abstract

Proton minibeam radiation therapy (pMBRT) is a novel strategy which has already shown a remarkable reduction in neurotoxicity as to compared with standard proton therapy. Here we report on the first evaluation of tumor control effectiveness in glioma bearing rats with highly spatially modulated proton beams. Whole brains (excluding the olfactory bulb) of Fischer 344 rats were irradiated. Four groups of animals were considered: a control group (RG2 tumor bearing rats), a second group of RG2 tumor-bearing rats and a third group of normal rats that received pMBRT (70 Gy peak dose in one fraction) with very heterogeneous dose distributions, and a control group of normal rats. The tumor-bearing and normal animals were followed-up for 6 months and one year, respectively. pMBRT leads to a significant tumor control and tumor eradication in 22% of the cases. No substantial brain damage which confirms the widening of the therapeutic window for high-grade gliomas offered by pMBRT. Additionally, the fact that large areas of the brain can be irradiated with pMBRT without significant side effects, would allow facing the infiltrative nature of gliomas.

## Introduction

Radiotherapy (RT) has a key role in cancer treatment. In fact, about half of the patients will receive RT at some point during their illness^1^. The therapeutic use of ionizing radiation has been largely guided by the goal of directly eliminating all cancer cells while minimizing the toxicity to adjacent tissues^2,3^. Nowadays, technological advances in radiation delivery, including image guidance and particle therapy (i.e. proton therapy), have notably improved tumor dose conformation, thus reducing the dose to the organs-at-risk^2,3^. However, the treatment of some radioresistant tumours, tumours close to a sensitive structure (e.g. central nervous system (CNS)) and pediatric cancers is still compromised due to the tolerances of normal tissues. However, the evolving wealth of biological knowledge^4–10^ allows seeking for new approaches. Indeed, the central dogma of conventional RT, namely that the cytotoxic effects of radiation are primarily due to the production of DNA double-strand breaks followed by some form of cell death (apoptosis, necrosis, autophagy, senescence), is being abandoned. This paradigm shift is led by the recent shreds of evidence of the importance of cell signaling^7,8^ and the role of the vascular^9^, stromal and immunological changes^10^ induced by the radiation in treatment outcome. The impact of those “non-targeted” effects on the biological response to radiation starts to be considered as a target itself to improve the therapeutic index in RT^4–10^.

Along this line, the utilization of distinct spatial distributions, such as in microbeam^11^ and minibeam radiation therapy (MBRT)^12,13^, seems to activate different biological mechanisms from those involved when direct damage by ionizing radiation takes place. There are indications of the activation of the immune system^14^ and cell signaling effects^15,16^. Another player appears to be the preferential effect on the tumoral versus normal vasculature. Normal tissues seem to benefit from the so-called microscopic prompt tissue-repair effect^15,17^, leading to a fast repair of vascular damage. In contrast, tumors presented a denudation of capillaries accompanied by transient increase in permeability and a decrease in the number of tumor vessels^17^. MBRT uses a combination of spatial fractionation of the dose and submillimetric (500–700 μm) field sizes^12,13^: the irradiation is performed by using an array of parallel thin beams spaced by 1 to 3 mm. The dose profiles in MBRT consist of peaks and valleys in contrast to the flat profiles in standard RT. Low energy X-rays MBRT notably increases normal tissue resistance in animal experiments^12,18–20^, while delaying tumor growth^21^. However, the need of using short-penetrating kilovoltage beams (to maintain the peaks and valleys pattern) and the lack of a hospital-based facility currently limit possible patients’ treatments in the coming years.

To profit from the benefits of MBRT, we have recently proposed to explore the synergies of proton therapy and the spatial fractionation of the dose^22^. This novel therapeutic approach is called proton minibeam radiation therapy (pMBRT). Proton MBRT offers several advantages over x-rays MBRT. First, a negligible dose is deposited in normal tissues after the Bragg peak (tumour position), further reducing the secondary effects. In addition, the multiple Coulomb scattering of protons allows obtaining a homogeneous dose distribution in the tumour with only one array of proton minibeams^22^. This contrasts with x-rays MBRT, in which two orthogonal arrays need to be used, leading to a more complex and error prone irradiation geometry. Moreover, recent studies have evidenced distinct biological properties of protons^23^.

We implemented this technique at the Orsay proton therapy center in 2014^24^. Dilmanian *et al*.^25^ confirmed the physical feasibility of the technique in 2015, in parallel to our first implementation^24^. A first study reporting on reduced side effects in the mouse ear after pMBRT with 20 MeV protons was published by a German team in 2016^26^. Even though the beam energy is not clinically relevant (a 20 MeV proton beam only penetrates 1 mm in the body), this work provided another indication of the advantages of this approach.

We have recently demonstrated that this technique notably increases the dose of tolerance of normal rat brain^27^. In a first experiment, the whole brain of two groups of normal rats was irradiated: *i)* a first group received a seamless (standard) proton irradiation, with high doses (25 Gy in one session); *ii)* a second group received pMBRT irradiation with peak doses of 58 Gy (corresponding to an average dose of 25 Gy). The animals were followed for up for 6 months. Rats treated with conventional proton irradiation exhibited severe moist desquamation and substantial brain damage. In contrast, no significant damage was observed in the pMBRT group^27^. This finding may improve the therapeutic index in cases with good rates of tumor control but accompanied of substantial side effects. It also opens the door for a more efficient treatment of very radio-resistant tumors, such as high-grade gliomas (GBM), still one of the most challenging cases in clinical oncology. The goal of this work was to perform a first evaluation of gain in the therapeutic index provided by pMBRT in the treatment of gliomas with highly spatially modulated dose distributions.

## Materials and Methods

All animal experiments were conducted in accordance with the animal welfare and ethical guidelines of our institutions. They were approved by the Ethics Committee of the Institut Curie and French Ministry of Research (permit no. 6361-201608101234488). Rats were anaesthetised with isoflurane (2.5% in air) during irradiation and magnetic resonance imaging (MRI). At the end of the study, the rats were terminally anaesthetised for brain fixation by the intracardiac perfusion of formalin zinc.

### Tumor cell line and tumor implantation

A rat glioma cell line, RG2-[D74] (ATCC^®^ CRL-2433^™^), was used. RG2 tumour model is known to be very aggressive *in vivo*, non-immunogeneic, with a highly invasive growth pattern, similar to human glioblastoma multiforme^28^. In particular, RG2 cells transfected with the luciferase gene were used to perform Bioluminescence Imaging (BLI) at an IVIS spectrum (PerkerElmer). BLI offers a simple and rapid technique for assessing intracranial glioblastoma growth in rodent models noninvasively^29–31^ and was therefore used to confirm the tumor presence before irradiation.

A number of 5000 RG2-Luc cells were suspended in 5 µl DMEM and then injected intracraneally into 19 Male Fischer 344 rats (Janvier Labs) using a Hamilton syringe through a burr hole in the right caudate nucleus (5 mm anterior to the ear-bars, i.e. at the bregma site, 3.0 mm lateral to the midline, and 5.5 mm depth from the skull). As average we have an implantation success rate of 97.8%.

For the BLI procedure the rats are injected intraperitoneally with a concentration of 150 mg/kgr (P/N 122799) of D-luceferin (Perkin Elmer) in 500 µl. The peak of luminescence is reached 25 minutes after injection. The presence of tumor is confirmed when the bioluminescent signal overcomes the background level. Thus, only the rats expressing a BLI signal significantly higher than the background on the day of the irradiation were included in the study. Table 1 shows the BLI signal values for those animals. The other rats were disregarded for further analysis.

**Table 1:** BLI values for the rats included in the study.

Rat	BLI signal after background subtraction (p/s)	Group
1	2,6 × 10^7^	control
2	6,9 × 10^6^	control
3	9,6 × 10^6^	control
4	5,7 × 10^7^	control
5	1,2 × 10^8^	control
6	9,7 × 10^6^	control
7	1,8 × 10^5^	control
8	7,3 × 10^7^	Irradiated
9	4,9 × 10^6^	Irradiated
10	5,0 × 10^7^	Irradiated
11	4,5 × 10^7^	Irradiated
12	2,5 × 10^7^	Irradiated
13	3,7 × 10^7^	Irradiated
14	1,5 × 10^7^	Irradiated
15	9,5 × 10^7^	Irradiated
16	8,6 × 10^7^	Irradiated

### Irradiations and dosimetry

The irradiations were performed at one of the horizontal beamlines (passive scattering) at the ICPO-Orsay with a proton beam energy of 100 MeV. This energy allows the treatment of a tumour located at the centre of the human brain (i.e., the worst scenario). This setup enabled the lateral irradiation of rat brains in the plateau region. The dose rate was 2 Gy/min at a 1-cm depth.

For minibeam generation, a multislit brass collimator was employed (400-μm-wide slits, 3200-μm centre-to-centre distance^24^) and was positioned 7 cm away from the rat skin. With this configuration, the minibeam width at a 1.0-cm depth was 1100 ± 50 μm. Further details of the experimental dosimetry in water phantoms can be found elsewhere^24^. Thus, large areas of the tumor received low doses (valley doses). Gafchromic films placed laterally on each side of the rat’s head (beam entry and exit) and attached to the skin allowed assessment of the quality of the irradiation.

Since the goal of this work was to evaluate the widening of the therapeutic window in pMBRT, two different set of experiments were performed. The first one aimed at evaluating the tumour control effectiveness, thus tumour-bearing rats were irradiated and compared to controls. The second one was a long-term evaluation of normal tissue toxicity, so normal rats (non-injected with any vehicle) were irradiated. Four groups of animals were considered: *i)* a control group (tumor bearing rats, non-irradiated) (n = 7); *ii)* a group of tumor bearing rats that received pMBRT 70 Gy peak dose at 1 cm depth, corresponding to an average of 30 Gy (n = 9). The irradiations were performed 8 days after implantation. No comparison with conventional (seamless) PT has been performed since such high mean doses would not be tolerated^27^. *iii)* a third group of normal (no-tumor implanted) rats (n = 9) that received pMBRT with the same dose and configuration than group *ii)*; *iv)* a second control group of normal rats (n = 5). All the doses were delivered in one fraction to avoid any possible blurring inter-fraction of the minibeam pattern due to positioning errors.

In all four groups of animals, 7 weeks-old rats at the moment of irradiation were considered. This fits within the range of age used in the clear majority of rats’ studies (between 6 and 8 weeks after birth). The reason is that the incidence of successful development of the tumor after implantation is much superior than at older ages, probably related to a less developed immune system at the younger age^32,33^. To the best of our knowledge there are not data to be able to infer where the tumor response to treatment would differ if old rats are used. Additionally, this age (7 weeks-old) was also convenient to evaluate the side effects in the developing (more sensitive) brain at long-term.

**Figure 1:**
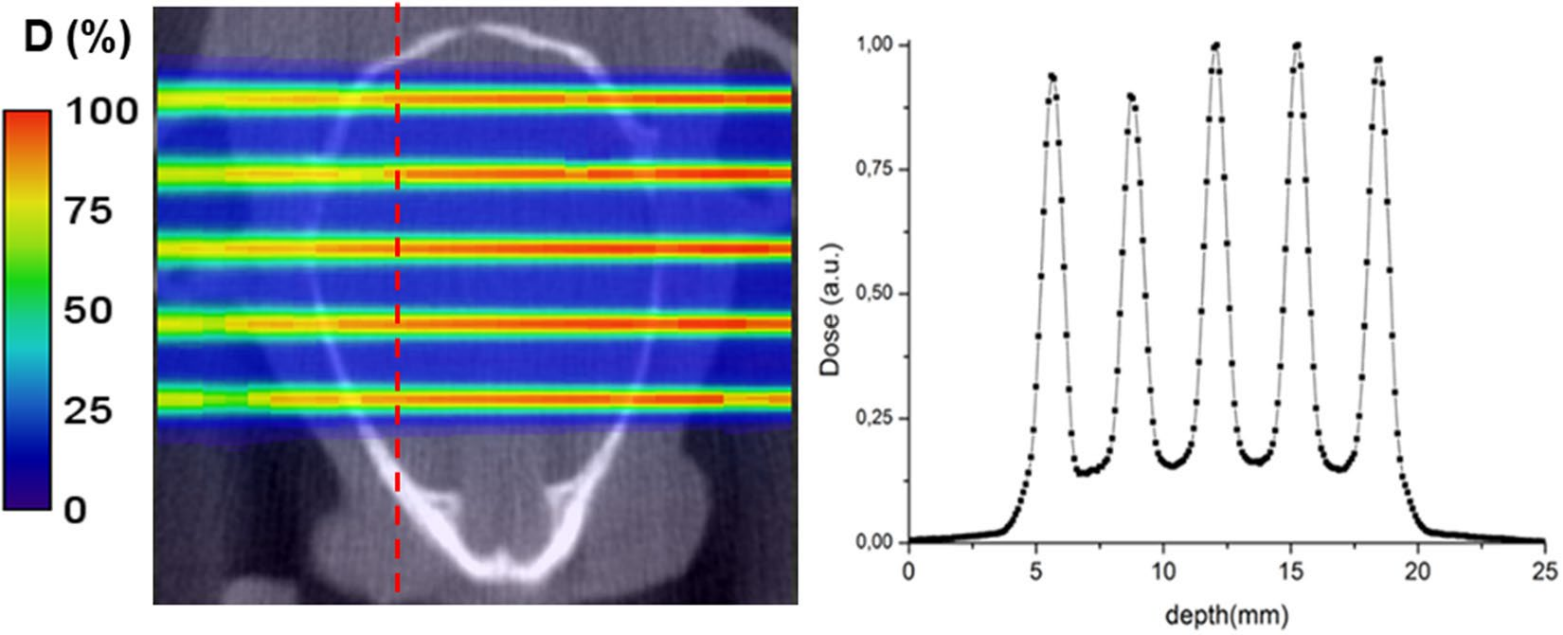
Dosimetry. Left: 2D dose map in the rat head (coronal view). The red line marks the approximate position of the center of the tumor. Right: Corresponding lateral dose profile at the tumor position.

To assess the dose distributions within the rats’ brains more precisely, Monte Carlo simulations (GATE v7.0) were employed. The whole proton beamline and the irradiation setting were modeled. Dose distributions were calculated in the computer tomography images of one of the representative rats, with a voxel scoring size of 1 mm × 100 µm × 2 mm in the vertical, lateral and beam directions respectively. Figure 1 illustrates how the tumor was irradiated with a highly heterogeneous dose distribution. It shows a 2D map of the dose distributions inside a rat’s head (left) and lateral dose profile at the expected position of the tumor (right). The PVDR values amount 6.1 ± 0.2 and 6.5 ± 0.2 at the expected tumor position (center) and at the middle of the brain, respectively.

### Animals follow up

The animals were followed-up for a maximum of 6 and 12 months in the case of tumour-bearing rats and normal rats, respectively. The clinical status of the animals was checked 5 and 2 times per week, in tumour bearing and normal rats, respectively. In the first case, any rat showing the classical adverse neurological signs related to the tumour growth in the brain was humanely killed. These signs could be any of the following: loss of appetite and substantial weight loss (>10% of the weight in 24 h), periorbital haemorrhages, seizures or prostration. To assess the survival curves, the median survival time post-implantation was calculated and Kaplan Meier survival data^34^ were plotted versus time after tumour implantation. The survival curves were compared using the log-rank test between the irradiated group and the controls (Prism-GraphPad).

In normal rats, the appearance of possible signs of distress was observed. Those include lack of grooming, hyperreactivity, apathy, spontaneous sound vocalizations, troubles of movement among others. In case of sustained weight loss (>20% of the animal maximal weight), ataxia, prostration, troubles of movement, seizures or periorbital haemorrhages, the animals would be humanly killed.

An anatomical MRI study was performed at 6 months and one year after irradiation in 5 out the nine animals in the normal rats’ series. Additionally, a MRI evaluation was also carried out in five irradiated tumour-bearing rats at 10 days after irradiation and in all long-term survivals (6 months after irradiation). For each imaging session, a catheter was inserted into the tail vein for contrast agent administration. A 7-Tesla preclinical magnet (Bruker Avance Horizontal 7-T Bruker, Inc., Billerica, MA) equipped with a 35-mm-diameter “bird-cage” antenna was employed. Three series were acquired:

i) Morphological T2-weighted (T2W) images with a repetition time (TR) of 2500 ms, an echo time (TE) of 33 ms, an echo spacing 11 ms, rare factor 6 a signal average of 2. In all, 21 slices were acquired.
ii) T1-weighted (T1W) TurboRare sequences with a TR of 800 ms and a TE of 6.05 m. A signal averaging of 2 was employed. A total of 21 slides were acquired. Three acquisitions were performed, one before and two (1.3 and 8 min) after the intravenous injection of a bolus of 100 µmol/kg Gd-DOTA (Guerbet SA, Villepinte, France).
iii) T1 fast low-angle shot (FLASH) sequences with a TR and TE of 114.89 and 3.1 ms, respectively. A flip angle of 30° and a signal averaging of 4 were used. A total of 9 slides were acquired in a total time of 1 min 28 s. Acquisitions were made just before and immediately and 6.30 min after the intravenous injection of a bolus of 100 µmol/kg Gd-DOTA (Guerbet SA, Villepinte, France).

All experiments were acquired in axial orientation. The field of view was 35 mm × 35 mm, the in-plane resolution amounted to 0.137 mm × 0.137 mm, and the slice thickness and gap were 0.8 and 0.3 mm, respectively.

As explained before the tumor-bearing rats were sacrificed when the endpoints related to tumor growth were reached or, in their absence, at 6 months after irradiation. Irradiated normal rats were sacrificed 6 months (n = 5) and 12 months (n = 4) after irradiation and compared to control rats (n = 4; no tumor and no irradiation), to evaluate the possible long-term side effects at different times. In all cases, the rats were terminally anaesthetised for brain fixation by the intracardiac perfusion of a fixative solution (formalin zinc). The brains were then removed, fixed in the fixative solution, and embedded in paraffin; 4-μm-thick sections were cut and stained in haematoxylin and eosin (HE) for the histopathological (double-blinded) analysis, carried out by ECVP (European College of Veterinary Pathologists) board certified pathologist. Immunohistochemistry analysis was performed to assess the networks and cell morphologies of microglia (anti-Iba-1 antibody, Wako Chemicals, dilution: 1:500) and astrocytes (anti-GFAP antibody, Sigma-Aldrich, dilution: 1:500). Astrocyte and microglial cell morphology are indeed directly linked to their physiological state^35^. These specific immunohistochemistry analyses allow detection of cell processes, *i.e*. visualization of the cell organization in space. Neuroinflammation is characterized by an activation of microglial cells: proliferation, thickening and shortening of the cell processes. Destruction of the neuropil and nervous tissue lead to an activation of astrocytes: proliferation, increase number and thickness of their cell processes. Ever increasing evidence support the hypothesis that late radiation-induced brain injury, including cognitive impairment, is driven by acute and chronic oxidative stress and inflammatory responses^36^. Table 2 summarizes the study groups and the corresponding follow-up.

## Results

This section reports on the tumor control effectiveness of pMBRT as well as on the long-term side effects of irradiated rats.

### Tumor control effectiveness evaluation

**Figure 2:**
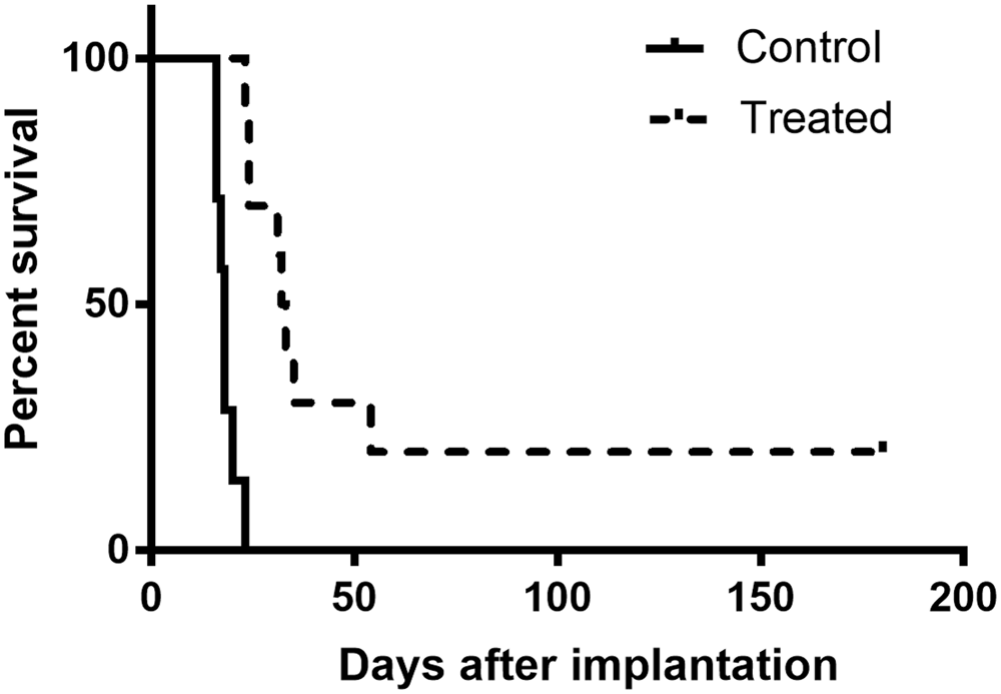
Survival curves for the controls (n = 7) versus pMBRT irradiated tumor-bearing rats (n = 9). pMBRT significantly increases tumor control. Additionally, a 22% of long-term survivals were obtained.

Figure 2 shows the survival curves of tumor bearing rats. The two curves showed to be statistically significantly different (p < 0.0001).

The mean survival time of the controls was 18 days in contrast to 32.5 days for the irradiated tumour-bearing animals. Two irradiated animals (number 10 and 11 in Tables 1 and 2) lived for the entire duration of the study and were then censored. Tumour sterilisation in these two long-term survivals was confirmed by the histology. It should be stressed that tumour eradication was achieved even with a highly heterogeneous dose distribution deposited in one fraction. In contrast, standard radiotherapy uses a homogeneous dose coverage of the target with the aim of depositing a lethal dose in every tumour cell. The prescribed dose is mostly deposited in several fractions.

**Table 2:** Study groups and follow-up.

Study groups	Sacrificed	MRI	Histology
Tumor bearing- rats, non-irradiated controls (n = 7)	When endpoints reached	No	All
Tumor bearing- rats, pMBRT (n = 9)	When endpoints reached or at the end of the study (6 months)	10 days after irradiation (n = 5) Long-term survivals (n = 2/9)	All
Normal rats, pMBRT (n = 9)	If endpoints reached or at the end of study: 6 months (n = 5) 12 months (n = 4)	6 months (n = 5) 12 months (n = 4)	All
Normal rats, controls (n = 4)	At the end of study	No	All

**Figure 3:**
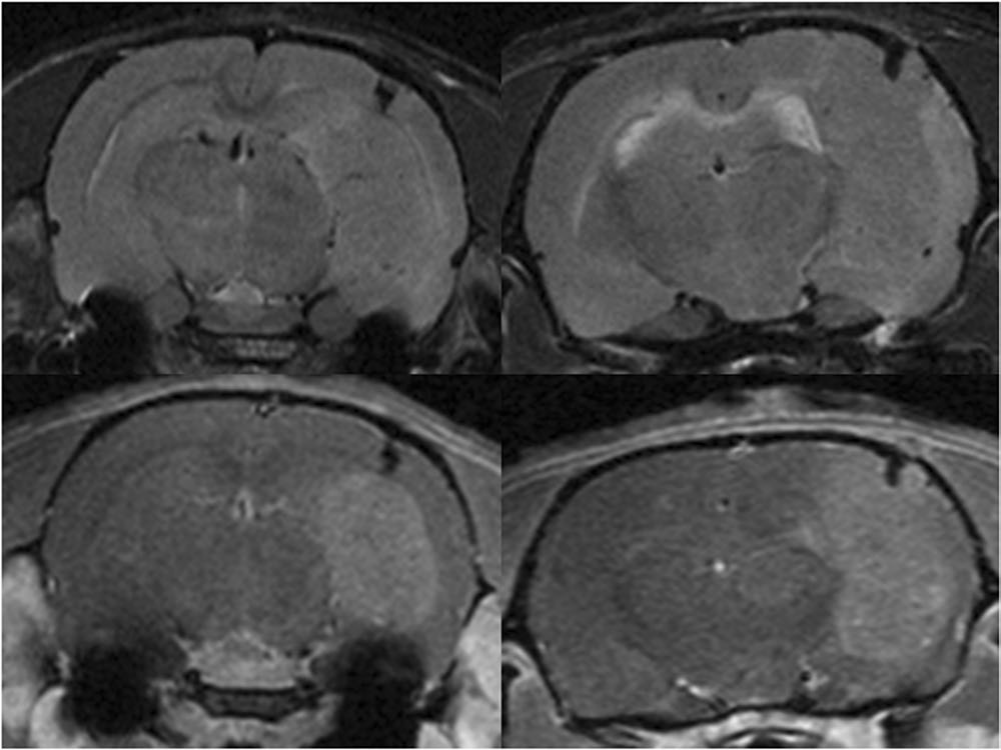
MRI evaluation at short-term. MRI images of two irradiated tumour-bearing rats taken 10 days after treatment. Upper row: T2w images. Lower row: T1w images 8 min after Gd injection. A large tumour in the right hemisphere is testified by a mass deforming the brain structures in T2w and a large area of blood barrier breakdown in T1w.

**Figure 4:**
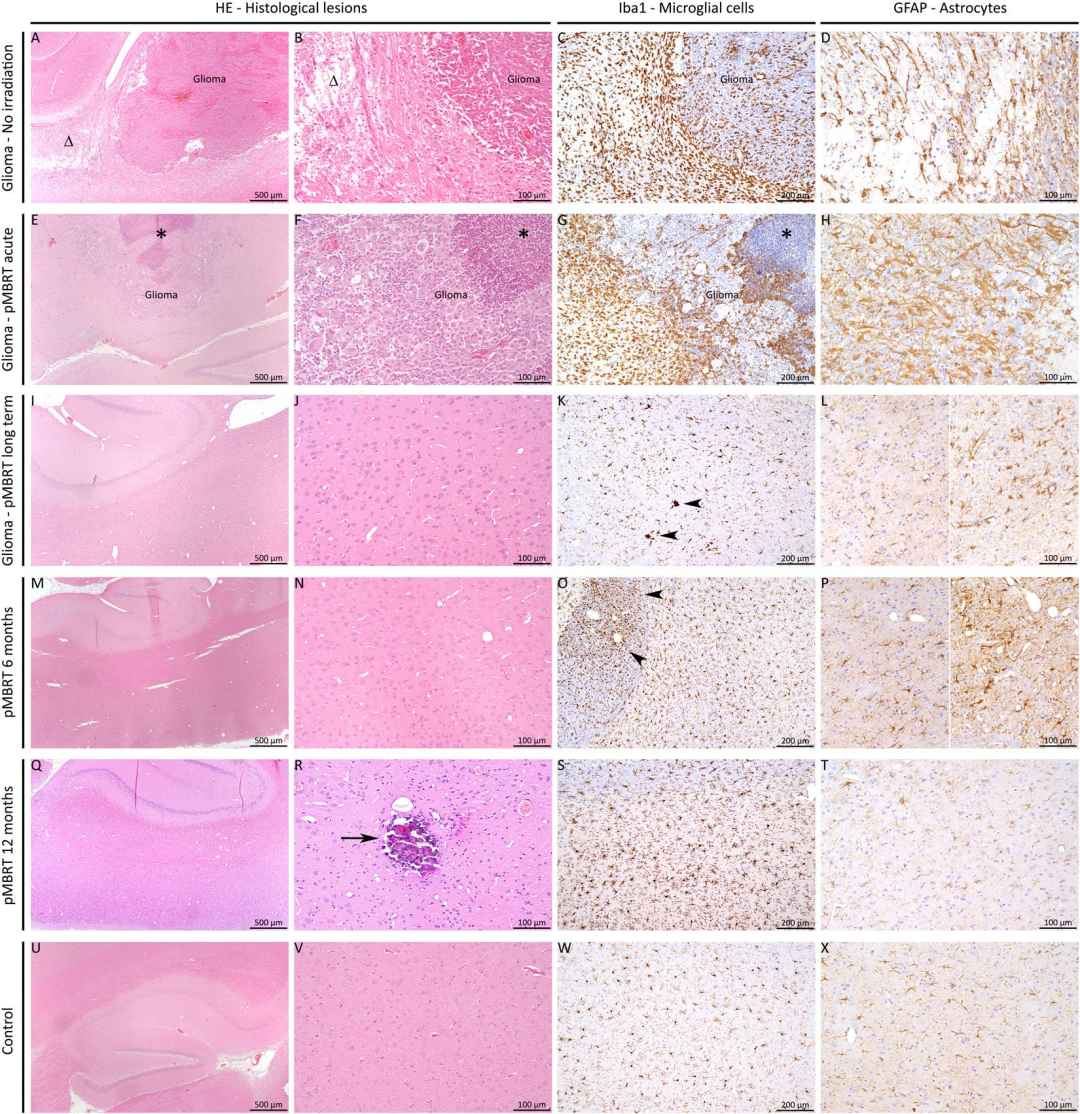
Histology. pMBRT irradiation protocol: strong impact on glioma but low impact on the normal brain tissue. (**A**–**D**) Without irradiation, rats displayed large gliomas (**A**), sometimes with peripheral necrosis (**A** and **B**, Δ) and proliferation/activation of microglial cells (thickening and shortening of their cell processes) in the tumor as well as at the periphery (**C**). In the necrotic areas, activation of astrocytes (proliferation and hypertrophy of cell body and processes: astrogliosis) was detected (**D**). (**E**–**H**) After pMBRT, for rats who had to be sacrificed at an early stage (less than 3 months), multifocal necrotic foci could be detected in the tumor (**E**–**G**, star) and brain tissue at the periphery, associated with a proliferation/hyperplasia of microglial cells (**G**) and astrocytes (**H**). (**I**–**L**) Long-term consequences of pMBRT were minimal on the brain tissue. Almost no lesion was indeed detected (in HE: **I**,**J**), except rare activated microglial cells (**K**, arrowheads), and astrocytes (**L**; left panel normal, right panel cluster of activated astrocytes). (**M**–**P**) 6 Months after irradiation, almost no lesion was detected in irradiated normal rats (**M**,**N**) except rare foci of microglial cell (**O**, arrowheads) and astrocyte (**P**; left panel normal, right panel cluster of activated astrocytes) activation. (**Q**–**T**) 12 Months after irradiation, the profile was similar as after 6 months, i.e. almost no lesion (**Q**) except moderate microglial cell hyperplasia (**S**) or foci of activation, associated with focal calcifications (**R**, arrow) in 2 rats. No astrogliosis was detected (**T**). (**U**–**X**) Control rats (no irradiation and no tumor) did not display any histological lesion (only one rat displayed small, randomly distributed calcification foci, data not shown). (**A**,**B**,**E**,**F**,**I**,**J**, **M**,**N**,**Q**,**R**,**U**,**V**) HE staining; (**C**,**G**,**K**,**N**,**R**,**V**) Iba1 immunohistochemistry to assess microglial cell density and morphology; (**D**,**H**,**L**,**P**,**T**,**X**) GFAP immunohistochemistry to assess astrocyte density and morphology.

Non-irradiated tumour-bearing rats displayed large gliomas at the moment of the sacrifice, sometimes with peripheral necrosis and hyperplasia/activation of microglial cells in the tumour as well as at the periphery. The pMBRT-irradiated rats who had to be sacrificed before 3 months after irradiation due to symptoms of tumour growth, presented very large gliomas and multifocal necrotic foci in the tumour and brain tissue at the periphery, associated with an activation/hyperplasia of microglial cells. See Figs 3 and 4. In brain tumor and brain metastasis, microglial cells and macrophages are recruited either within or near the tumor masses. Some works suggests that they might play an important role in brain tumor progression^35^.

**Figure 5:**
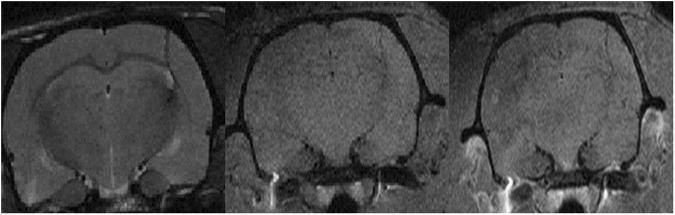
MRI evaluation of long-term survivals. Upper row: T2w (left), T1w before (center) and 8 min after Gd injection (right) images of one of the long-term survivals. No substantial damage was observed with the exception of a scar in the tumor injection site. No tumor presence was observed.

Concerning the long-term survivals neither substantial damage nor tumor presence were observed in the MRI images nor in the histopathological analysis. Only minimal lesions, as small clusters of astrocyte and microglial cell activation (minimal neuroinflammation) were found. See Figs 4 and 5.

### Normal rats’ irradiations: evaluation of long-term side effects on normal tissues

The irradiated normal rats gained weight normally. No external clinical symptoms were noted. No skin damage was developed. A reversible epilation in the minibeam path was observed.

**Figure 6:**
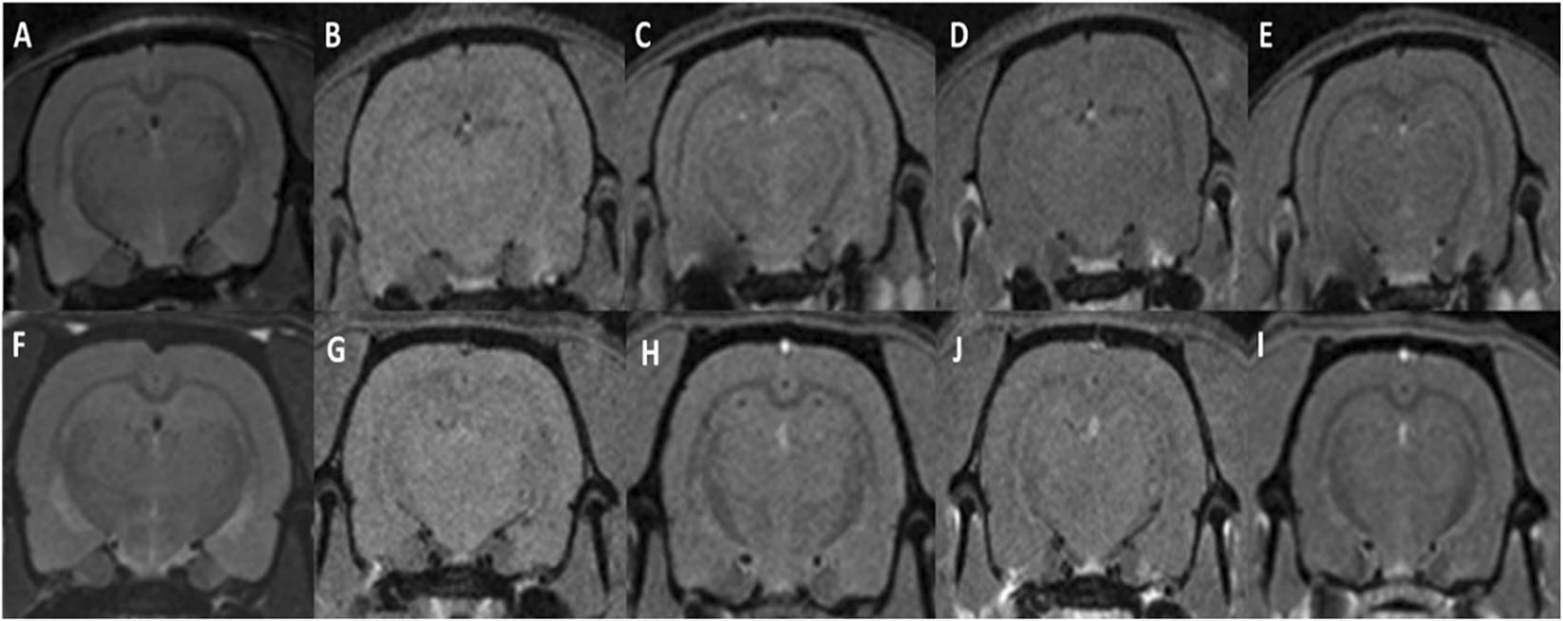
MRI evaluation of normal rats. Comparison of the MRI images acquired at 6 months after irradiation for one of the irradiated animals (upper row) versus one of the controls (lower row). T2w images (**A**,**F**), RARE-T1w images before (**B**,**G**) and after 8 min Gd injection (**D**,**J**) and FLASH-T1w images before (**C**,**H**) and after (**E**,**I**) Gd injection are shown. No tissue damage is observed and no significant difference was found between irradiated and control animals.

Six months after irradiation 5 irradiated normal animals were imaged and compared to the healthy controls. No damage was observed, no significant differences with the non-irradiated controls were found. See Fig. 6.

The histopathological analysis performed 6 months after irradiation revealed only minimal lesions: rare foci of microglial cell and astrocyte activation. No significant necrosis nor neuropil destruction were observed. See figure 4.

**Figure 7:**
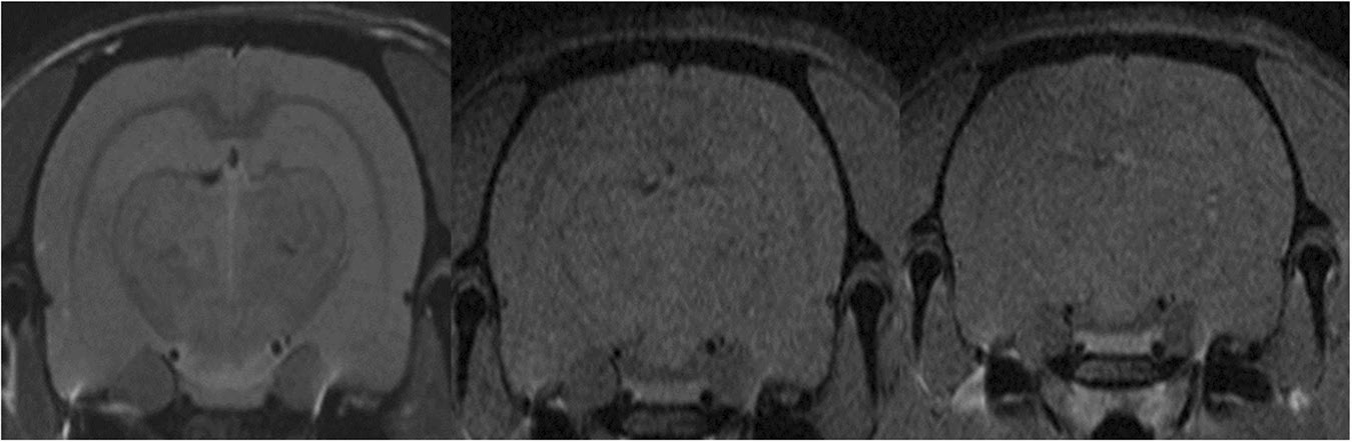
MRI acquisition of irradiated normal rats after one year of follow up. T2w (left) and T1w-RARE images before (center) and 8 minutes (right) after Gd injection. No substantial lesions are observed.

In the 4 rats that were followed up for one year, the profile was similar to the group sacrificed at 6 months, *i.e*. no visible lesion in the MRI analysis (see Fig. 7) and almost no lesion detected in the histological evaluation (except rare foci of microglial cell and astrocyte activation and neuropil mineralisation). No necrosis, neuropil destruction, or astrogliosis were observed (Fig. 4).

## Discussion

Human glioblastoma multiforme is one of the most aggressive tumours with a median survival of 7–15 months from the time of diagnosis. The gold standard management of GBM, tumour resection followed by radiotherapy and chemotherapy (typically temozolomide), is limited in efficacy due to high rates of recurrence, overall resistance to therapy, and devastating side effects^37^. Therefore, finding alternative efficient solutions is of utmost importance. This work is the first evaluation of the increase in therapeutic index provided by pMBRT in the treatment of high-grade gliomas with very heterogeneous dose distributions. Whole brain irradiations excluding the olfactory bulb were performed with peak doses of 70 Gy at 1 cm-depth, corresponding to a mean dose of 30 Gy. The spacing between the beams was 3.2 mm. Thus, large areas of the tumour receive (low) non-lethal doses. No comparison with conventional (seamless) PT has been performed since such high mean doses would not be tolerated^27^. In our previous work^27^ severe damage, including radionecrosis was observed in the group of rats that received 25 Gy in conventional proton therapy. These results are coherent with some other studies using X-rays^38^. To the best of our knowledge there are not *in vivo* preclinical evaluations of the effects on the brain of conventional temporally fractionated schemes in proton therapy to compare with. One of the main reasons is the impossibility of employing several hours of beamtime of a clinical proton beamline daily. The availability of experimental proton beamlines would make it feasible. However, in brain patients, treated with conventional several fractions-schemes in proton therapy, the percentage of induced radionecrosis is high and it can reach 50% in pediatric patients^39^.

pMBRT has shown a very significant effectiveness of tumour control in RG2 glioma bearing rats, achieving as well a 22% of long-term survivals, free of tumour. And that, without some of substantial side effects, such as radionecrosis, that would have been observed with standard PT, already at lower doses^27,39^, thus opening the possibility for even more aggressive irradiation schemes. Further evaluations on neurogenesis integrity and behavioural tests to discard any cognitive impairment will be performed. The optimization of the irradiation parameters, such as the beam spacing or the dose, might further increase the number of curations in pMBRT.

Long-term survivals were also obtained in gliosarcoma (9 L) bearing rats treated with very heterogeneous dose distributions in microbeam radiation therapy (MRT)^14,40,41^. A direct comparison with this study is not possible due to the different cell lines employed. However, this work shows that similar results could obtained with pMBRT using a dose 4 times lower than in MRT. Enormous doses of more than 300 Gy in the peaks, around 18 Gy valleys and 120 Gy mean dose, are needed in MRT to obtain a significant tumour control. The fact of using supra-millimetre minibeams (1.1 mm at 1-cm depth) removes the need of very high dose rates to avoid the beam smearing of cardiosynchronous pulsations in MRT^42^. In addition, the implementation and dosimetry are technically easier and much less error prone when thick beams are used. Finally, in contrast to MRT, our studies in pMBRT are directly performed at a clinical centre, which makes the transfer to potential clinical trials a direct one.

It is worth noting that tumour control and tumour sterilization were achieved with such heterogeneous dose distribution in one fraction. In contrast, the classical paradigm of RT requires the deposition of a lethal dose in every tumour cell for tumour ablation. The dose is usually delivered in several fractions to reduce normal tissue toxicity and favour tumour re-oxygenation.

Our hypothesis to explain our results with such distinct dose delivery is the participation of some non-targeted effects. Indeed, temporal schemes using very high-dose radiation in one fraction have been reported to transform the immunosuppressive tumour microenvironment resulting in an intense CD8 T-cell tumour infiltrate^14,43^. Some hints of the participation of cell signalling effects^15,16^ have been reported in other spatially fractionated techniques. Another possible player might appear to be the preferential effect on the tumoral versus normal vasculature^15,17^. The investigation of the possible participation of all those phenomena requires a comprehensive approach, and it is out of the scope of this work.

The study on normal rats confirms a remarkable tissue-sparing effect of 70-Gy supra-millimetre minibeams and relatively low PVDR (around 6.5). These results have important implications for potential clinical trials, since it opens the door for future implementations with pencil beam scanning systems (millimetre-size beams). The significant increase in tumour control effectiveness and the reduced neurotoxicity observed in the irradiated normal rats confirms the widening of the therapeutic window. Moreover, the fact that large areas (or even whole brain) could be irradiated without significant side effects, could allow overcoming one of the major difficulties to be faced in gliomas treatment: its infiltrative nature. One of the key problems when treating patients with malignant gliomas is that, despite being able to remove the major bulk of the tumor through neurosurgery, it is known that malignant tumor cells have already spread throughout the brain and even extensive resections will not cure the patient.

Finally, the results of this study show that pMBRT could offer a completely innovative and promising way of using protons for therapy. Moreover, the lack of need of a homogeneous coverage of the target to achieve tumour control along with the reduced toxicity in the surrounding normal tissues diminish the requirements in terms of positioning and ballistics precision. This could optimize the patient workflow, reducing proton therapy costs consequently.

## Data Availability

The datasets generated and analysed during the current study are available from the corresponding author on reasonable request.
